# Glial Cytokine and Metabolic Networks in Progressive Multiple Sclerosis: From Pathophysiology to Biomarkers and Therapeutic Strategies

**DOI:** 10.3390/ijms26188817

**Published:** 2025-09-10

**Authors:** Henry Leonard Hohm, Rasmus Schuster, Victor Bogdan Buciu, Denis-Mihai Serban, Sebastian Ciurescu, Amalia Cornea, Abhinav Sharma, Daciana Nistor, Nilima Rajpal Kundnani

**Affiliations:** 1 Faculty of Medicine, “Victor Babes” University of Medicine and Pharmacy Timisoara, E. Murgu Square, No. 2, 300041 Timisoara, Romania; henryhohm@gmail.com (H.L.H.); raschu1911@gmail.com (R.S.); 2 Doctoral School, “Victor Babes” University of Medicine and Pharmacy Timisoara, E. Murgu Square, No. 2, 300041 Timisoara, Romania; sebastian.ciurescu@umft.ro (S.C.); sharma.abhinav@umft.ro (A.S.); 3 Department of Obstetrics-Gynaecology, Discipline of Obstetrics-Gynecology, “Victor Babes” University of Medicine and Pharmacy Timisoara, E. Murgu Square, No. 2, 300041 Timisoara, Romania; denis.serban@umft.ro; 4 Department of Neurosciences, Neurology II Division, Victor Babeș University of Medicine and Pharmacy, 300041 Timișoara, Romania; amalia.cornea@umft.ro; 5 University Clinic of Internal Medicine and Ambulatory Care, Prevention and Cardiovascular Recovery, Department VI-Cardiology, “Victor Babes” University of Medicine and Pharmacy, 3000041 Timisoara, Romania; 6 Department of Functional Sciences, Physiology, Center of Immuno-Physiology and Biotechnologies (CIFBIOTEH), “Victor Babes” University of Medicine and Pharmacy, 300041 Timisoara, Romania; 7 Centre for Gene and Cellular Therapies in Cancer, 300723 Timisoara, Romania; 8 Research Centre of Timisoara, Institute of Cardiovascular Diseases, “Victor Babes” University of Medicine and Pharmacy, 3000041 Timisoara, Romania

**Keywords:** progressive multiple sclerosis, cytokines, neuroinflammation, glial activation, oxidative stress, astrocyte

## Abstract

Progressive multiple sclerosis (PMS) represents a distinct clinical and biological entity characterized by compartmentalized neuroinflammation, chronic glial activation, and resistance to conventional immunotherapies. Unlike relapsing MS, PMS is sustained by resident CNS immune networks, where activated microglia and astrocytes orchestrate persistent cytokine signaling—particularly involving TNF-α, IL-1β, and IL-6—through self-amplifying feedback loops. In this narrative review, we explore how these cytokines interact with oxidative stress, iron accumulation, mitochondrial dysfunction, and impaired autophagy to drive neurodegeneration. Human-based evidence is integrated with insights from experimental models to clarify translational mechanisms. We also highlight fluid biomarkers (e.g., GFAP, NfL) and imaging modalities (e.g., TSPO-PET, QSM) that reflect glial activity and disease progression in vivo. Age, sex hormones, and immunosenescence are discussed as modulators of cytokine expression. Finally, we review emerging therapeutic strategies that target glial metabolism and cytokine networks rather than peripheral immune cells, offering a systems-based framework for future PMS interventions and personalized disease monitoring.

## 1. Introduction

Multiple sclerosis (MS) is an immune-mediated disorder of the central nervous system (CNS) characterized by chronic inflammation, demyelination, gliosis, and axonal degeneration. It is one of the most common causes of neurological disability in young adults, affecting over 2.8 million people worldwide [1,2]. The disease manifests in various clinical forms, with the most common being relapsing–remitting MS (RRMS), which may evolve into secondary progressive MS (SPMS). Primary progressive MS (PPMS) is a less common but more disabling form of the disease that lacks clinical relapses and exhibits steady neurological decline from onset [3].

Progressive MS (PMS), encompassing both SPMS and PPMS, is distinguished by its lack of response to conventional immunomodulatory therapies. This discrepancy is largely attributed to the shift from peripheral immune infiltration observed in RRMS to compartmentalized inflammation within the CNS in PMS [4,5]. In this context, the resident immune cells—particularly microglia and astrocytes—assume a central role in disease progression [4,5,6]. They maintain a pro-inflammatory environment through chronic cytokine production, which exacerbates neurodegeneration by disrupting homeostatic glial functions, impairing mitochondrial activity, and perpetuating oxidative stress [4,6].

The persistent activation of glial cells and the resulting neuroimmune signaling cascades are now recognized as fundamental drivers of PMS pathogenesis [6,7]. Cytokines, small signaling proteins secreted by immune and glial cells, are key mediators of this process [7,8]. Their complex roles in cell communication, immune recruitment, and inflammatory regulation are both protective and pathological, depending on the temporal and spatial context [8]. Understanding their specific contributions in PMS is essential for identifying novel biomarkers and designing targeted therapeutic interventions [6,7]. Throughout this review, findings derived from animal models (e.g., EAE, cuprizone, lysolecithin) are explicitly identified, while evidence from human studies is clearly specified as originating from clinical observations, post-mortem tissue analysis, or biomarker investigations.

This review aims to provide an in-depth analysis of the cytokine landscape in PMS. We begin by outlining the altered neuroimmune architecture characteristic of PMS, then systematically examine individual cytokines and inflammatory mediators implicated in disease progression. By integrating mechanistic data, comparative pathology, and therapeutic insights, we propose a comprehensive framework for rethinking cytokine-targeted strategies in progressive MS.

### What Is New in This Review

The present review advances beyond recent syntheses on glial pathology in PMS by integrating cytokine signaling networks—including TNF-α, IL-6, IL-1β, IL-10, TGF-β, and GM-CSF—with key metabolic and structural processes such as mitochondrial dysfunction, iron dysregulation, and autophagy failure into a unified framework of disease progression [6,9]. Unlike earlier works that have examined these elements in isolation [10,11,12] [10,11], we explicitly stratify the evidence according to study type (human in vivo, human post-mortem, animal models, in vitro), thereby clarifying the translational weight of mechanistic versus correlative findings. Furthermore, this review connects glial cytokine patterns to validated and emerging biomarkers—GFAP and NfL in biofluids, and TSPO-PET and QSM for imaging—outlining how these markers can guide patient stratification and endpoint selection in PMS clinical trials [13,14]. We also incorporate the most recent therapeutic data, including the Phase 3 HERCULES trial of tolebrutinib in non-relapsing SPMS [15], and updated evidence from TSPO-PET longitudinal studies [14,16], QSM lesion profiling [9,17], and metabolic or iron-targeting strategies [18,19], positioning these findings within a glia-centric translational strategy for PMS.

This review builds on previous work by linking cytokine signaling (including TNF-α, IL-6, IL-1β, IL-10, TGF-β, and GM-CSF) with core metabolic and structural changes such as mitochondrial dysfunction, iron dysregulation, and autophagy failure to present a comprehensive model of disease progression in PMS [6,9].

To contextualize the scope and novelty of this review, Table 1 contrasts its focus, biomarker coverage, and therapeutic discussion with those of recent high-quality syntheses on glial pathology in progressive multiple sclerosis (PMS) published between 2024 and 2025.

Rather than proposing a single new hypothesis, this review offers a systems-based synthesis that explicitly integrates cytokine signaling with metabolic stressors (mitochondrial failure, iron dysregulation, impaired autophagy) into a unified framework of PMS progression, thereby clarifying mechanistic interdependencies and their biomarker correlates.

## 2. Review Methodology

### 2.1. Aim of the Study

The aim of this review was to synthesize current knowledge on the role of glial-derived cytokines in the pathogenesis of PMS, with emphasis on the molecular mechanisms driving neuroinflammation, oxidative damage, and therapeutic resistance. Special attention was given to the interplay between tumor necrosis factor-alpha (TNF-α), interleukin-6 (IL-6), and interleukin-1β (IL-1β), and their downstream mediators—including inducible nitric oxide synthase (iNOS), cyclooxygenase-2 (COX-2), mitochondrial stress, iron accumulation, and impaired autophagy. In addition, the review sought to highlight translational biomarkers and emerging therapeutic strategies targeting glial activation in PMS.

### 2.2. Literature Search Strategy

A structured literature search was carried out across three major databases—PubMed/MEDLINE, Web of Science Core Collection, and Scopus—to identify high-quality, peer-reviewed studies published between January 2000 and June 2025. The search was designed to retrieve mechanistic, translational, and clinical evidence relevant to glial cytokine signaling in PMS.

Keywords and Boolean operators were tailored to include a combination of disease-specific, molecular, and therapeutic terms. The following search string was used with minor database-specific adaptations: (“progressive multiple sclerosis” OR “PMS”) AND (“cytokines” OR “TNF-alpha” OR “IL-6” OR “IL-1β”) AND (“microglia” OR “astrocytes” OR “glial activation”) AND (“oxidative stress” OR “autophagy” OR “mitochondria” OR “NLRP3”) AND (“biomarkers” OR “TSPO-PET” OR “GFAP” OR “NfL” OR “iron” OR “ferroptosis”) AND (“treatment” OR “therapeutic strategies” OR “glial modulation”).

Search filters were applied to include only English-language articles published in ISI-indexed journals. Additional studies were identified through manual examination of reference lists from key reviews and landmark articles.

The final database update was completed on 15 June 2025.

### 2.3. Eligibility Criteria and Selection Process

To determine which studies would be included in this review, we applied a set of predefined eligibility criteria based on relevance to the core objectives. Articles were considered eligible if they explored glial cytokine expression or signaling specifically within the context of progressive multiple sclerosis. We included studies that examined the role of microglia or astrocytes in driving neuroinflammation and neurodegeneration, as well as those that described downstream pathological mediators—such as nitric oxide, reactive oxygen species, mitochondrial dysfunction, or impaired autophagy.

Further inclusion criteria extended to studies evaluating biomarkers of glial activity, either through fluid-based assays (e.g., cerebrospinal fluid, serum) or neuroimaging techniques such as TSPO-PET or susceptibility-weighted MRI. Lastly, we included experimental or clinical studies that proposed or investigated therapeutic strategies aimed at modulating glial pathways, cytokine activity, or associated metabolic dysfunction. Only studies that met at least one of these criteria and were published in peer-reviewed, ISI-indexed journals were selected for final inclusion. Articles were excluded if they focused exclusively on relapsing–remitting MS, discussed peripheral immunomodulation without relevance to CNS-compartmentalized inflammation, lacked mechanistic insight, or were conference abstracts, editorials, or non-peer-reviewed sources.

A comprehensive literature search was conducted across PubMed, Web of Science, and Scopus to identify relevant publications on progressive multiple sclerosis. Priority was given to peer-reviewed studies focusing on central nervous system pathology, glial mechanisms, biomarkers, and therapeutic strategies. The evidence base was then synthesized to develop the mechanistic overview, biomarker discussion, and therapeutic framework presented in this manuscript. All selections were reviewed by two independent authors, and disagreements were resolved through discussion and consensus.

In order to enhance transparency and facilitate interpretation of the evidence base, all included studies were classified into four broad categories according to their methodological approach and translational relevance. These are summarized in Table 2, which outlines the evidence type, key strengths, guidance for integration, and modes of bias for each category. This tiered framework was applied as a fit-for-purpose quality assessment tool, helping to distinguish mechanistic insights from correlative associations and to indicate the level of readiness for clinical translation.

### 2.4. Rationale for Narrative Review Format

Although the literature on PMS-related cytokines is expanding, it remains methodologically diverse. The included studies varied substantially in design, ranging from molecular and cellular models to histopathological studies, neuroimaging analyses, biomarker validation, and early-phase therapeutic trials. Due to this high degree of heterogeneity, a meta-analytic or quantitative synthesis was not feasible. Instead, the decision was made to conduct a narrative review, which allows for integrative analysis and conceptual organization across disparate but complementary data sources. This approach is particularly suited to the systems-level nature of PMS pathogenesis, which cannot be adequately captured by isolated metrics or effect sizes.

This review is structured as a narrative review with systematic search elements to ensure comprehensive literature coverage. We have included a PRISMA flow diagram to transparently illustrate the search and selection process; however, the review does not meet all methodological criteria for a full systematic review (e.g., formal risk-of-bias scoring). Instead, our approach is designed to synthesize and critically interpret the existing evidence base.

### 2.5. Quality Assurance and Reporting Standards

To ensure scientific rigor, only articles published in ISI-indexed, peer-reviewed journals were included. These criteria served as a proxy for minimum methodological quality. Although we did not perform formal risk-of-bias assessments or apply grading tools such as GRADE, we prioritized studies with reproducible experimental methods, translational relevance, and clearly defined CNS-specific findings. The review was structured in line with the SANRA (Scale for the Assessment of Narrative Review Articles) guidelines, emphasizing clarity of objectives, methodological transparency, justified article selection, and coherent synthesis of the evidence.

Given the narrative scope of this review, we did not apply a formal risk-of-bias tool (e.g., Cochrane Risk-of-Bias, PEDro scale) to the included studies. Instead, we prioritized the inclusion of research from ISI-indexed, peer-reviewed journals with clear methodological descriptions, which served as a pragmatic quality filter. While a structured bias appraisal was outside the intended scope, future syntheses aiming for meta-analytic integration should incorporate standardized quality assessments to further strengthen the robustness of conclusions.

Throughout this review, we distinguish between correlative associations and mechanistic evidence, noting when findings derive from human in vivo, post-mortem, animal, or in vitro studies to clarify translational strength.

## 3. The Neuroimmune Landscape of PMS

PMS is marked by a shift in the inflammatory milieu from peripheral lymphocyte-driven responses, typical of RRMS, to a more insidious form of inflammation that is compartmentalized within the CNS [23,24], while the blood–brain barrier (BBB) remains relatively preserved [25]. This ‘smoldering’ inflammation is sustained by innate immune responses, particularly involving microglia and astrocytes, which undergo phenotypic and functional changes in response to prolonged inflammatory stimuli [4,23,26].

Microglia, the resident immune cells of the CNS, transition into a chronically activated state in PMS (in human autopsy tissue). These cells exhibit increased expression of activation markers such as Iba1 (ionized calcium-binding adaptor molecule 1) and CD68, a lysosomal protein involved in phagocytic activity [23,26]. Rather than adopting the classical M1/M2 polarization seen in peripheral macrophages, PMS-associated microglia display a complex, disease-associated phenotype that includes features of both inflammation and phagocytosis [23,24,26,27]. This phenotype contributes to sustained cytokine release, oxidative damage, and synaptic stripping.

**Figure 1:**
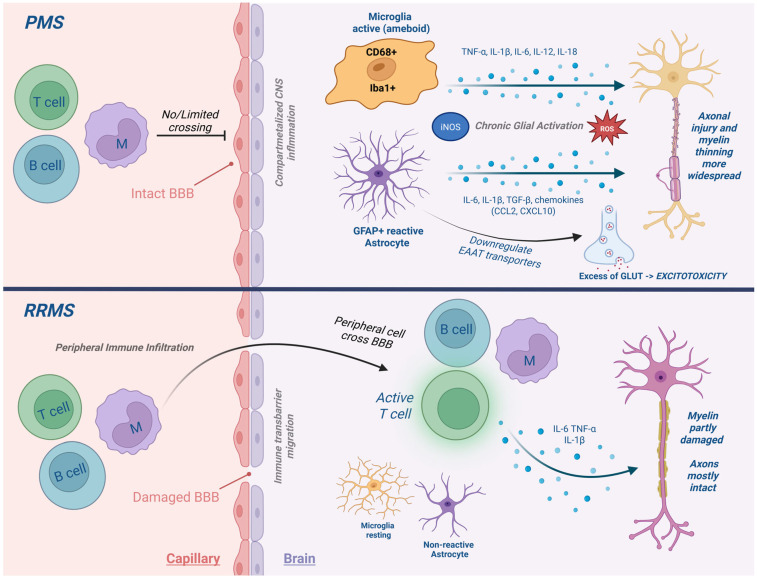
Neuroimmune cell activation in PMS. Comparison of immune activity in relapsing versus progressive MS. In PMS, compartmentalized inflammation is sustained by activated microglia and reactive astrocytes, leading to chronic cytokine release, oxidative stress, and damage to the BBB.

Astrocytes, traditionally considered as supportive glial cells, also play a pivotal role in perpetuating neuroinflammation (Figure 1). Reactive astrocytes form glial scars (shown in both EAE models and PMS autopsy studies), secrete pro-inflammatory cytokines (e.g., IL-6, IL-1β), and contribute to excitotoxicity through dysregulation of glutamate uptake. Their interactions with microglia and endothelial cells reinforce the inflammatory microenvironment and compromise the blood–brain barrier (BBB) [28,29,30].

In PMS, the inflammatory profile shifts from peripheral lymphocyte-driven infiltration, characteristic of RRMS, to a more insidious, compartmentalized form of inflammation within the CNS, despite relative preservation of the BBB [25]. Astrocytes play a central role in this process: reactive astrocytes, documented in both PMS autopsy studies and EAE models, form glial scars, release pro-inflammatory cytokines (IL-6, IL-1β), and exacerbate excitotoxicity via impaired glutamate uptake. Through their crosstalk with microglia and perivascular cells, astrocytes sustain the chronic intrathecal inflammatory milieu that drives neurodegeneration in progressive disease.

A hallmark feature of PMS is the presence of slowly expanding, chronic active lesions with a hypocellular core surrounded by a rim of activated microglia. These lesions exhibit mitochondrial dysfunction and progressive axonal loss. Importantly, iron distribution is heterogeneous: iron tends to accumulate at the rim of smoldering lesions, while levels are reduced in the lesion center, and overall iron concentrations are often lower compared with normal-appearing white matter [31,32]. This nuanced pattern underscores the complex role of iron in sustaining microglial activation and oxidative stress in PMS. Imaging studies using PET ligands targeting translocator protein (TSPO) have confirmed the presence of widespread microglial activation even in normal-appearing white matter (NAWM) and gray matter (GM) [8,26,33,34,35].

All aforementioned glial markers of progressive MS, their function and relevance towards the disease are described in Table 3.

Notably, cytokine expression patterns can vary across PMS phenotypes. SPMS often shows elevated CSF IL-6 and CXCL13 levels, particularly in patients with meningeal inflammation, while PPMS more frequently displays sustained TNF-α expression and greater iron-rich microglial activation in deep gray matter. These differences may influence both biomarker profiles and therapeutic responsiveness, underscoring the need for phenotype-tailored intervention strategies.

Understanding the neuroimmune architecture of PMS is essential for interpreting cytokine dynamics, as these molecules act within a compartmentalized and chronically inflamed environment that is fundamentally distinct from the relapsing form of MS.

## 4. Key Cytokines and Inflammatory Mediators in PMS

In PMS, the inflammatory landscape diverges significantly from that of relapsing disease, marked instead by a sustained, compartmentalized immune response centered within the central nervous system [26,29,36]. At the core of this process are resident glial cells—microglia and astrocytes—that become chronically activated and secrete a constellation of cytokines, perpetuating a cycle of immune signaling, oxidative damage, and neurodegeneration [7,26,36].

Among the most central mediators in this cytokine network are tumor TNF-α, IL-6, and IL-1β, each of which plays a critical role in sustaining glial reactivity. TNF-α, largely produced by activated microglia and astrocytes, exerts its pathogenic effects predominantly through TNFR1, a receptor that promotes apoptosis, blood–brain barrier disruption, and oligodendrocyte injury [37,38,39]. Although TNFR2 signaling is thought to exert neuroprotective effects, its activation appears underrepresented in PMS, which may contribute to disease persistence. Elevated TNF-α expression has been consistently observed in chronic active lesions of PMS patients (human post-mortem tissue), suggesting its prominent role in ongoing inflammation [37,38].

IL-6, another key cytokine largely of astrocytic origin, amplifies neuro-inflammatory responses by activating the STAT3 signaling pathway [40,41,42]. This leads to widespread astrogliosis and contributes to cortical demyelination, particularly in secondary progressive MS where it has been associated with meningeal inflammation. Clinical studies have demonstrated increased IL-6 levels in the cerebrospinal fluid of PMS patients (clinical biomarker studies), often correlating with progressive cortical thinning and gray matter atrophy [40,41,42,43,44].

Interleukin-1β, in turn, is primarily activated through the NLRP3 inflammasome and serves as a potent upstream driver of both TNF-α and IL-6 production [6,45,46,47]. This establishes a tightly coupled feedback system, in which IL-1β sustains glial activation while fueling the transcription of other pro-inflammatory mediators. Both human post-mortem tissue and rodent models of progressive MS reveal persistent NLRP3 expression, underscoring its importance in chronic disease pathology [46,47].

In addition to these pro-inflammatory mediators, anti-inflammatory and growth-factor cytokines influence PMS pathology. IL-10, secreted by microglia, astrocytes, and regulatory lymphocytes, inhibits NF-κB activation and shifts glial cells toward reparative phenotypes; its reduced expression in PMS lesions compared with RRMS may reflect an impaired resolution response. TGF-β generally suppresses inflammation via Smad signaling, yet chronic overexpression in PMS has been linked to astrocytic scar formation and extracellular matrix deposition. GM-CSF, produced by astrocytes and CNS-infiltrating T cells, drives pathogenic microglial activation and has been associated with cortical demyelination in both EAE and PMS autopsy cohorts [48,49].

The downstream consequences of this cytokine milieu extend well beyond immune signaling. Inducible iNOS, induced by chronic cytokine exposure, facilitates the production of nitric oxide, which impairs mitochondrial respiration and damages synaptic structures. In parallel, the upregulation of COX-2 fosters prostaglandin synthesis, further contributing to neuroinflammation and excitotoxicity [50,51,52]. Reactive oxygen species (ROS), particularly those arising in iron-rich microglia, amplify tissue injury through lipid peroxidation and DNA damage, compounding the destructive effects of chronic inflammation [36,51].

Complicating these processes is the consistent disruption of autophagy observed in PMS. Markers such as LC3 and p62 reveal impaired autophagic flux in affected brain regions, indicating that damaged mitochondria and aggregated proteins are not being adequately cleared [53,54,55]. This inefficiency feeds back into inflammasome activation and perpetuates cellular stress responses, further entrenching glial dysfunction [53,54,55,56,57].

Together, these cytokines and mediators do not operate in isolation but constitute an interdependent network that drives disease progression. Understanding their individual roles and collective dynamics provides critical insight into both the molecular underpinnings of PMS and the therapeutic opportunities that may arise from targeting glial signaling hubs. Table 4 summarizes the major cytokines and associated mediators in PMS, outlining their primary CNS sources, downstream effects, and potential therapeutic targets.

To increase transparency, we provide an evidence-tier map (Table 5) that categorizes key cytokine claims by relative weight of human versus preclinical evidence, clarifying the basis of the proposed TNF-α/IL-6/IL-1β axis.

Taken together, the available evidence indicates that TNF-α, IL-6, and IL-1β form a tightly interconnected inflammatory axis that is consistently detected in human PMS autopsy tissue, CSF biomarker studies, and experimental models. While their roles in promoting glial activation and neurodegeneration are well-supported, variability exists in the magnitude and spatial distribution of cytokine expression across studies, possibly reflecting differences in PMS phenotypes, disease stage, or methodological approaches. The relative underrepresentation of anti-inflammatory mediators such as IL-10 and TGF-β in PMS lesions suggests an imbalance favoring chronic inflammation. Longitudinal human studies integrating cytokine profiling with imaging markers are needed to clarify temporal dynamics and causal relationships.

### Sex and Age-Related Modulation of Cytokine Activity in PMS

Cytokine profiles in progressive MS are shaped not only by cellular and molecular factors, but also by demographic variables such as sex, age, and hormonal status. Women generally exhibit more robust innate and adaptive immune responses, which may account for the earlier onset of relapsing MS. However, the female predominance narrows in PMS, particularly in primary progressive MS, where male patients exhibit faster neurodegenerative decline [58,59]. Estrogen and progesterone have been shown to suppress pro-inflammatory cytokines such as TNF-α and IL-1β, while enhancing IL-10 expression; their decline during menopause may partially explain the transition toward a pro-inflammatory profile in older women [60,61]. Conversely, aging is associated with immunosenescence, mitochondrial dysfunction, and a skewing toward low-grade chronic inflammation (“inflammaging”), characterized by elevated circulating IL-6, CRP, and TNF-α [58,59,60]. These shifts may intensify glial priming, oxidative damage, and autophagy impairment in PMS. Hormonal modulation of cytokine signaling, particularly through sex-steroid receptors expressed on microglia and astrocytes, represents a promising yet underexplored target for future therapeutic interventions [37,58,59,60,61].

Cerebrospinal fluid studies demonstrate that male PMS patients often exhibit higher IL-6 and CXCL13 concentrations than females [62]. In contrast, post-menopausal women show elevated TNF-α and reduced IL-10 levels [63], suggesting a loss of estrogen-mediated immunoregulation. TSPO-PET imaging indicates greater microglial activation in deep gray matter among male PPMS patients, correlating with faster disability accumulation [64].These findings highlight sex-linked differences in CNS innate immune activation that may inform personalized therapeutic strategies.

## 5. Cytokine Crosstalk and Disease Progression

The pathological environment of progressive multiple sclerosis is shaped not by isolated cytokine actions but by a complex web of interactions between various inflammatory mediators and cellular stress pathways. This “crosstalk” forms a self-perpetuating cycle of glial activation, oxidative stress, mitochondrial dysfunction, and neurodegeneration. In this network, cytokines such as TNF-α, IL-6, and IL-1β act not merely in parallel, but in concert, synergizing through feedback loops that sustain chronic inflammation [7,23,26,28,29,30,39,45,52].

**Figure 2:**
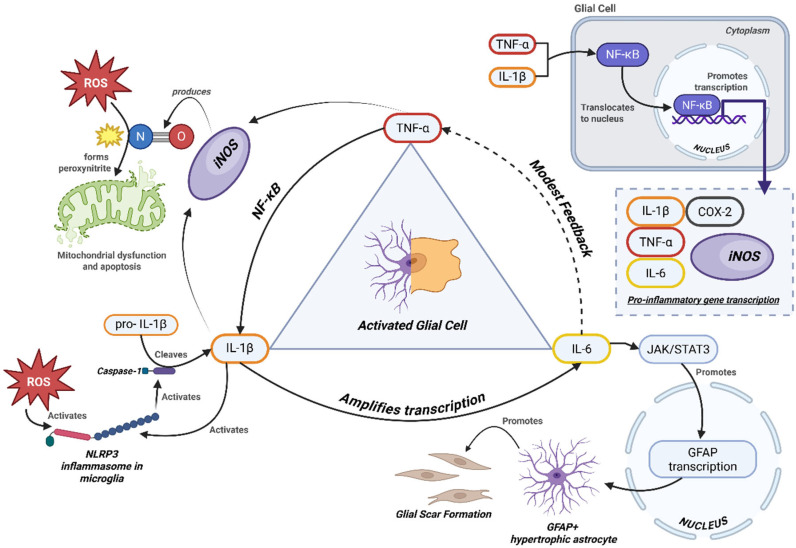
Cytokine feedback network in progressive MS.

The dynamic interplay of these cytokines, oxidative mediators, and stress pathways in PMS is illustrated in Figure 2.

Integrated schematic of cytokine-driven pathways contributing to neuroinflammation in PMS. Pro-inflammatory cytokines (TNF-α, IL-6, IL-1β) activate downstream mediators (iNOS, ROS), mitochondrial stress, and impaired autophagy. Reactive astrocytes (GFAP+) and NG2 glia form the structural core of the glial scar, with contributions from fibroblast-like cells. This barrier restricts axonal regrowth while perpetuating cytokine release and oxidative stress [65,66].

The pathological environment of progressive multiple sclerosis is driven not by isolated cytokines, but by a synergistic network in which TNF-α, IL-6, and IL-1β perpetuate inflammation through interconnected feedback loops. As detailed in Section 4, these cytokines activate shared transcriptional pathways (e.g., NF-κB, STAT3) and reinforce each other’s expression through glial crosstalk [6,37,46,50]. This cytokine synergy sustains a pro-inflammatory, metabolically stressed glial state that drives neurodegeneration.

Key downstream effectors—such as iNOS, COX-2, and ROS—amplify the damage by promoting oxidative stress, mitochondrial dysfunction, and autophagy impairment. In turn, these stressors reactivate inflammasomes like NLRP3, forming a vicious cycle of immune activation and cellular injury [36,51].

This interconnected inflammatory axis suggests that single-cytokine inhibition may be insufficient for disease modification in PMS. Instead, targeting nodal points in the cytokine network—such as inflammasomes, metabolic checkpoints like AMPK, or iron homeostasis—may prove more effective [23,36,56]. Understanding this crosstalk is also vital for biomarker development, as measuring isolated cytokine levels may miss the broader inflammatory context. These converging molecular loops highlight the systems-level nature of PMS pathology and underscore the need for interventions that address multiple interacting pathways rather than isolated targets.

In addition to their mechanistic roles, these glial and metabolic changes are increasingly detectable through biofluid analysis and advanced neuroimaging techniques [33,46].

### Biomarkers and Imaging in Progressive Multiple Sclerosis

The growing recognition of progressive MS as a compartmentalized, CNS-centric inflammatory disease has prompted efforts to identify reliable biomarkers that reflect glial activation, cytokine dysregulation, and neurodegeneration. These markers are essential not only for diagnosis and prognosis, but also for tracking treatment response in emerging network-level therapeutic strategies.

One of the most promising fluid biomarkers is glial fibrillary acidic protein (GFAP), which reflects astrocytic reactivity and is significantly elevated in the cerebrospinal fluid (CSF) and serum of patients with secondary and primary progressive MS [67,68]. GFAP levels have shown correlation with spinal cord atrophy and disease progression. While GFAP is strongly associated with non-relapsing progression and disability accrual in PMS, its long-term prognostic precision remains uncertain. GFAP dynamics may be influenced by co-existing pathologies such as small vessel disease or other neurodegenerative processes. Future biomarker strategies should integrate GFAP with complementary measures, including NfL and advanced neuroimaging modalities such as TSPO-PET, to improve predictive accuracy.

In large PMS cohorts, serum GFAP predicted disability progression with HR 1.45–1.78 per SD increase, corresponding to AUC values of 0.68–0.73 for ≥3-year progression [13]. Sensitivity and specificity at optimized thresholds typically range 65–75%. Compared with EDSS progression, GFAP captures astroglial injury more specifically but is less sensitive to relapse-related activity. By contrast, NfL shows higher sensitivity (AUC 0.78–0.82) but poor specificity, underscoring its role as part of a composite panel [68].

Another well-established marker, neurofilament light chain (NfL), reflects axonal injury and is now widely used in clinical trials as a general indicator of neurodegeneration, though it lacks specificity for glial-driven processes [46,69,70]. NfL is a robust biomarker of neuroaxonal injury and correlates with disease activity and progression in PMS. However, it lacks specificity for glial-driven processes, as elevated levels are also observed in other neurological conditions such as Alzheimer’s disease, Parkinson’s disease, and amyotrophic lateral sclerosis. Consequently, its interpretation in PMS should be made in conjunction with glia-specific biomarkers such as GFAP or TSPO-PET.

Among microglial markers, soluble TREM2 (sTREM2) and CD14 are being evaluated as CSF markers of innate immune activation. Elevated CSF IL-6, IL-8, and CXCL13 levels have also been associated with intrathecal inflammation and cortical atrophy, providing insight into cytokine-mediated progression [8,71,72,73].

Neuroimaging complements these fluid markers by enabling spatial localization of neuro-inflammatory processes. TSPO PET imaging, using second-generation ligands, offers a noninvasive measure of glial activation in vivo [70,74,75,76]. Elevated TSPO signal in deep gray matter and periventricular white matter has been correlated with disability in PMS [7,33]. Interpretation of TSPO-PET findings must account for binding-affinity polymorphisms (e.g., rs6971) and the need for standardized acquisition and analysis protocols across centers. Without addressing these factors, cross-study comparability is limited, which should be considered in multicenter trial design. In prospective designs, genotype handling should stratify rs6971 binders (≈65% high, 30% mixed, 5% low affinity in European cohorts), with harmonized ligand selection, dynamic acquisition, and centralized quantification pipelines to ensure reproducibility across sites [21].

In parallel, quantitative susceptibility mapping (QSM) and iron-sensitive MRI techniques allow visualization of chronic active lesions, often characterized by paramagnetic rims linked to iron-retaining microglia [70,74,75,76].

Together, fluid and imaging biomarkers offer a systems-level approach to characterizing disease activity in PMS. Their integration into clinical trials and precision medicine protocols may enable stratification of patients based on inflammatory profile and guide personalized therapeutic interventions.

The summary of fluid and imaging biomarkers used to evaluate glial activation, neuroinflammation, and neurodegeneration in progressive multiple sclerosis can be found in Table 6.

**Figure 3:**
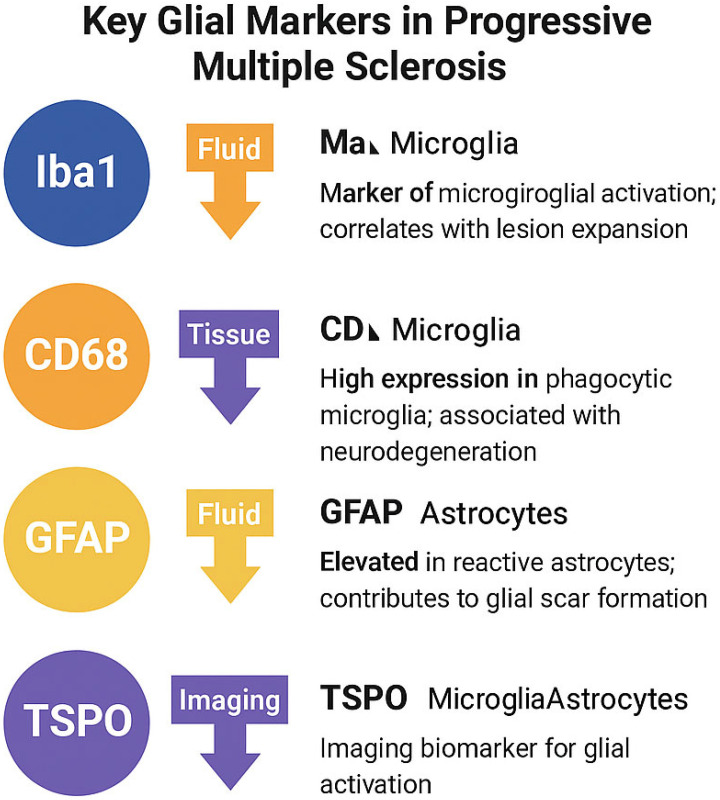
Summary of key glial markers in progressive multiple sclerosis. Iba1 and CD68 are associated with microglial activation and phagocytosis, while GFAP reflects reactive astrogliosis. TSPO is a mitochondrial translocator protein expressed in both activated microglia and astrocytes, and is widely used in PET imaging to quantify glial inflammation.

A consolidated overview of the most commonly used glial activation markers—based on cellular specificity, function, and relevance to PMS pathology—is presented in Figure 3.

Note: While TSPO expression is not exclusive to microglia, its overexpression in activated glial cells, particularly under neuro-inflammatory conditions, makes it a widely accepted imaging biomarker in PMS. Expression may also be influenced by genotype (e.g., rs6971 polymorphism) and cell type [77,78].

Overall, GFAP and NfL emerge as the most consistently validated fluid biomarkers in PMS, with robust associations with clinical progression and imaging metrics. However, they lack specificity for glial subtypes or precise mechanistic pathways. Microglial markers such as sTREM2 and CD14, while promising, require validation in larger longitudinal cohorts and standardization of assay methods. Few studies directly compare biomarker profiles between SPMS and PPMS, representing a critical gap for future research.

## 6. Therapeutic Implications and Future Directions

The complex interplay between cytokines, glial activation, oxidative stress, and autophagy dysfunction in PMS underscores the limitations of traditional immunosuppressive therapies. Agents targeting single cytokines—such as TNF-α inhibitors—have failed in clinical trials, largely due to redundancy within inflammatory networks and the opposing roles of receptor subtypes (e.g., TNFR1 vs. TNFR2) [39,79,80]. Several cytokine-targeted therapies have failed in PMS clinical trials; TNF-α inhibition with lenercept was associated with worsening disability [81], while ustekinumab (anti-IL-12/23) showed no efficacy in halting disability progression [82]. The main reasons include redundancy in inflammatory pathways, compensatory cytokine upregulation, and insufficient CNS penetration of large molecules. These lessons emphasize the need to target central network hubs such as inflammasomes, metabolic checkpoints, or iron metabolism rather than single cytokines [6]. A more effective strategy requires interventions that act on nodal points within these interlinked systems.

One such approach involves AMPK activation. Agents like metformin, a well-characterized metabolic modulator, restore autophagic flux, reduce inflammasome activation, and suppress IL-6/STAT3 signaling in glial cells. Preclinical studies in PMS models have shown that metformin improves mitochondrial efficiency and dampens microglial activation [54,83,84]. Its known safety profile and CNS penetrance have led to its repurposing in clinical trials for neurodegenerative diseases, including ongoing Phase II investigations in PMS [8,83,84]. Metformin remains an investigational therapy in PMS, with ongoing evaluation in the MACSiMiSE-BRAIN trial and inclusion within the OCTOPUS adaptive platform; efficacy results are pending [85].

Bruton’s tyrosine kinase (BTK) inhibitors, such as tolebrutinib, offer another promising avenue. These small molecules modulate B cell function and CNS-resident myeloid cells, including microglia [10,86]. Tolebrutinib has demonstrated CNS penetration and is currently in Phase III trials for both SPMS and PPMS, showing potential to attenuate glial-driven inflammation without broadly suppressing peripheral immunity [10,39]. The Phase 3 HERCULES trial, published in April 2025, demonstrated that tolebrutinib significantly reduced six-month confirmed disability progression in non-relapsing SPMS patients, representing the first positive late-phase result in this population [15].

Iron chelation therapy addresses a distinct pathological axis: oxidative stress driven by iron accumulation in microglia [87,88]. Agents like deferiprone mitigate ROS production by preventing Fenton chemistry, reducing mitochondrial injury and tissue damage [36,88,89]. Targeted CNS delivery remains a challenge, but early-phase studies suggest biologically meaningful iron load reduction with acceptable tolerability [87,88].

mTOR inhibition via agents such as rapamycin is also under investigation, particularly for its role in enhancing autophagy and modulating astrocyte reactivity. While mTOR inhibitors have shown neuroprotective effects in preclinical models, clinical trials in PMS are limited, and the immunosuppressive effects of systemic mTOR blockade remain a concern [56,90,91]. mTOR pathway modulation, including rapamycin, has shown benefit in preclinical EAE models, but evidence in human PMS remains limited, and extrapolation from animal data should be made cautiously [92].

Lastly, redox modulation represents an adjunctive therapeutic strategy. Nrf2 activators, such as dimethyl fumarate, enhance endogenous antioxidant responses, suppress ROS-mediated glial priming, and may complement metabolic- or autophagy-based interventions. Although approved for RRMS, their role in PMS remains under evaluation, especially in combination therapies [36,39,93].

Future success in PMS treatment will likely require multi-targeted or combination strategies that simultaneously address inflammation, metabolic collapse, and impaired proteostasis. A precision medicine framework, guided by CSF cytokine profiles, iron imaging (e.g., QSM MRI), or microglial activation markers (e.g., TSPO PET), will be critical for patient selection and therapeutic monitoring.

In summary, therapeutic innovation in PMS must move beyond linear, cytokine-specific approaches. Network-based interventions targeting glial metabolism, oxidative injury, and autophagic integrity offer a more mechanistically aligned and potentially transformative strategy for altering the natural course of this progressive and treatment-resistant disease.

A systems-level understanding of PMS pathogenesis has led to the emergence of novel therapeutic strategies aimed at modulating inflammation, metabolism, iron balance, and autophagy. Table 7 summarizes these agents, highlighting their molecular targets, mechanisms of action, and current stage of clinical development.

Despite encouraging preclinical and early clinical signals for agents such as metformin, tolebrutinib, and deferiprone, most candidate therapies have not progressed to late-phase PMS trials. This reflects the complexity and redundancy of the underlying cytokine networks, the challenge of achieving adequate CNS penetration, and the lack of validated biomarkers to guide patient selection. Adaptive trial designs and multi-targeted intervention strategies may help overcome these barriers.

## 7. Comparative Insights from Other Neurodegenerative Diseases

Many of the cytokines implicated in progressive multiple sclerosis are also involved in other chronic neurodegenerative disorders, including Alzheimer’s disease (AD), Parkinson’s disease (PD), amyotrophic lateral sclerosis (ALS), and stroke [92,93,94]. However, while molecular overlaps exist, the pathophysiological context and therapeutic responsiveness often differ substantially due to tissue-specific factors such as immune compartmentalization, glial priming, and metabolic state [1,89,94].

For example, IL-6 is prominently elevated in both PMS and AD, contributing to astrocyte activation and neurodegeneration. In both diseases, IL-6 is a driver of STAT3 signaling, making it a shared target for immunomodulation [53,94,95]. Similarly, COX-2 upregulation is observed in PMS, AD, and PD, where it is linked to glial inflammation and synaptic dysfunction [50,53,94]. These parallels support the rationale for repurposing drugs across disease models, although the chronicity and compartmentalization seen in PMS may limit efficacy unless agents can cross the blood–brain barrier and act on CNS-resident cells.

By contrast, TNF-α’s role in ALS differs from PMS in both source and effect. In ALS, peripheral macrophage infiltration contributes significantly to cytokine load, while PMS involves long-term microglial TNF-α production within the CNS [50]. This distinction underpins the limited success of anti-TNF therapies in PMS despite their broader immunosuppressive activity. Iron retention and ROS production are also seen in PD and ALS [46,68,80]. However, the chronic microglial iron loading and impaired efflux observed in PMS are relatively unique. While systemic iron chelation has shown benefits in ALS models, PMS may require targeted strategies that address CNS iron metabolism and mitochondrial vulnerability [51,87].

Lastly, autophagy impairment is a converging mechanism across PMS, PD, and AD. In all three diseases, the accumulation of LC3 and p62 reflects defective clearance of damaged proteins and organelles [51,52,53,96]. Pharmacologic agents such as metformin and rapamycin, which restore autophagy, show promise in preclinical models of each condition and may have broad neuroprotective potential [53,57].

Thus, while PMS shares multiple cytokine and metabolic features with other neurodegenerative diseases, its unique, CNS-confined, chronic inflammatory environment necessitates disease-specific strategies. Comparative insights can guide drug development, but therapeutic translation requires consideration of disease context, glial biology, and neuroimmune dynamics. We provide this comparative analysis in Table 8.

## 8. Conceptual Framework

Based on the evidence reviewed, we propose a network-level conceptual framework in which chronic activation of microglia and astrocytes sustains a self-amplifying cytokine circuit dominated by TNF-α, IL-6, and IL-1β, with modulatory input from IL-10, TGF-β, and GM-CSF [20,22]. These cytokines converge on oxidative stress pathways, iron dysregulation, and defective autophagy, maintaining a metabolically compromised glial phenotype [53,97]. Mitochondrial dysfunction and persistent inflammasome activation (e.g., NLRP3) reinforce cytokine production, while iron-rich microglia amplify ROS-mediated injury [46].

This integrated model differs from previous reviews by explicitly linking cytokine signaling to mitochondrial–iron–autophagy axes and by tiering the supporting evidence by study type (human in vivo, post-mortem, animal, in vitro). From a translational perspective, this framework aligns directly with multimodal biomarkers: GFAP and NfL in biofluids for glial injury and axonal loss [88], TSPO-PET for in vivo glial activation [14,21], and QSM detection of paramagnetic rim lesions (PRLs) for chronic active lesions [9].

Such alignment supports targeted patient enrichment in progressive MS trials, for example, prioritizing GFAP-high, TSPO-PET-positive, and PRL-positive individuals as “network-active” candidates for interventions targeting nodal points of the framework. These may include metabolic checkpoints (e.g., AMPK activation via metformin), iron chelation (e.g., deferiprone) [18], BTK inhibition (e.g., tolebrutinib; NEJM HERCULES trial, 2025) [15], or modulation of inflammasome activity. Embedding biomarker endpoints alongside clinical disability measures may improve both the mechanistic interpretability and the efficiency of PMS trials, moving beyond isolated cytokine blockade toward multi-axis glial network modulation.

### 8.1. Implications for Patient Stratification

We propose a pragmatic enrichment approach for PMS studies that uses GFAP-high (non-relapsing progression), TSPO-PET-high (innate glial activation), and QSM PRL-positive (iron-retaining rims) as orthogonal selectors to identify “network-active” patients most likely to benefit from glia-targeted interventions. Combining GFAP with NfL can help separate non-inflammatory progression from relapse-related activity and reduce misclassification [14].

### 8.2. Implications for Endpoints and Design

For trials targeting network nodes (e.g., BTK inhibition, metabolic/autophagy modulation), we suggest coupling clinical disability with biomarker endpoints: longitudinal GFAP/NfL (progression vs. degeneration), TSPO-PET (glial activation), and QSM PRLs (chronic activity). The recent HERCULES results for tolebrutinib illustrate that disability-progression endpoints can be met in non-relapsing SPMS; embedding TSPO/QSM as secondary endpoints may sharpen pharmacodynamic interpretation and responder profiling [14,15].

### 8.3. Key Messages for Clinicians

To improve clinical usability, we distilled the main findings of this review into a set of concise, practice-oriented statements. These “Key Messages” are intended as an at-a-glance guide for clinicians and researchers, highlighting the most robust evidence on cytokine networks, biomarkers, and therapeutic strategies in progressive multiple sclerosis. By summarizing complex translational data into clear take-home points, this section provides a bridge between mechanistic insights and day-to-day decision-making in both clinical care and trial design:

▪ Progressive MS is sustained by compartmentalized glial cytokine networks (TNF-α, IL-6, IL-1β).
▪ Evidence is strongest for TNF-α and IL-6 in human tissue and CSF; IL-1β remains largely model-driven.
▪ GFAP and NfL are validated progression biomarkers; combining them with TSPO-PET and iron-sensitive MRI improves specificity.
▪ TSPO-PET requires binder-genotype handling and standardized analysis.
▪ Iron rims on QSM MRI identify chronically active lesions driving progression.
▪ BTK inhibitors (tolebrutinib) show first late-phase efficacy in non-relapsing SPMS.
▪ Network-based interventions (AMPK activation, iron chelation, autophagy modulation) may outperform single-cytokine targeting.
▪ Patient enrichment using GFAP, TSPO-PET, and PRL markers may accelerate PMS trial success.

## 9. Conclusions

PMS is driven by a tightly interwoven network of glial-derived cytokines, oxidative mediators, and metabolic stress signals that together perpetuate CNS inflammation and neurodegeneration. Far from a linear autoimmune process, PMS emerges as a systems-level pathology in which TNF-α, IL-6, and IL-1β converge with mitochondrial dysfunction, iron dysregulation, and impaired autophagy to sustain a non-resolving glial phenotype. This review underscores the importance of shifting from single-target immunosuppression toward therapies that disrupt network hubs, such as the inflammasome, AMPK axis, or iron metabolism. Biomarker integration through CSF profiling, iron-sensitive MRI, and molecular imaging is poised to refine patient stratification and enable a precision medicine framework. By targeting the feedback architecture that underpins progressive disease, future strategies may finally alter the natural course of PMS.

The novelty of this review lies in its integrative framework that connects glial cytokine signaling with iron, mitochondrial, and autophagy pathways, while aligning these mechanistic axes with translational biomarkers and therapeutic strategies. This approach provides a conceptual blueprint for network-level interventions and personalized monitoring in PMS.

Ultimately, cytokines in PMS are not merely markers of inflammation but drivers of disease progression. Intervening in their network-level dynamics offers a mechanistically informed pathway toward halting or even reversing CNS damage in progressive MS.

## Figures and Tables

**Table 1:** Comparison of this review with recent high-quality syntheses (2024–2025) on glial pathology in progressive multiple sclerosis.

Year/First Author	Primary Focus	Biomarkers/ Imaging Discussed	Therapeutics Discussed	Distinctive Contribution of Present Review
2024—Garton et al. [20]	Immune–glia interactions in demyelination/degeneration with PMS focus	General biomarker overview; limited integration of iron, mitochondria, autophagy	Broad disease-modifying therapy overview	Mechanistic cell biology of glia; limited linkage between biomarkers and therapeutic targeting
2024—Voon et al. [9]	Quantitative susceptibility mapping (QSM) methodology and sensitivity to iron/myelin	QSM and paramagnetic rim lesions (PRLs)	None	Imaging physics and lesion detection; no cytokine network integration
2025—Guido et al. [21]	TSPO-PET in MS, validation, prognostic use	TSPO-PET longitudinal and standardization	None	Detailed TSPO-PET; lacks integration with cytokine/iron/autophagy axes
2025—Vermersch et al. [22]	Microglia roles and targeting in MS	Selected biomarkers; narrative emphasis	Microglia-targeted agents	Microglial biology; minimal biomarker-to-trial framework
Present review	Glial cytokine network + metabolic/structural axes in PMS	GFAP, NfL, TSPO-PET, QSM PRLs	BTK inhibitors (incl. HERCULES), metabolic modulation, iron chelation, mTOR targeting	Unified network linking cytokines ↔ mitochondria ↔ iron ↔ autophagy; explicit evidence tiering; trial-stratification blueprint

Abbreviations: BTK—Bruton’s tyrosine kinase; GFAP—glial fibrillary acidic protein; MS—multiple sclerosis; NfL—neurofilament light chain; PRL—paramagnetic rim lesion; QSM—quantitative susceptibility mapping; TSPO-PET—translocator protein positron emission tomography.

**Table 2:** Classification of included studies.

Study Category	Typical Sample Size	Strengths	Common Bias Modes	Integration Guidance
Human in vivo (CSF/imaging, clinical trials)	50–500	Direct clinical translation	Selection bias, medication confounding, disease-stage heterogeneity	Prioritize replicated CSF/imaging results; caution if small n or mixed phenotypes
Human post-mortem	10–80	High-resolution cellular detail	Terminal-stage bias, lack of longitudinal dynamics	Useful for mechanism; avoid overgeneralizing prevalence
Animal models (EAE, cuprizone)	8–30 per arm	Controlled mechanistic testing	Species differences, short disease course	Best for mechanistic plausibility; validate in human tissue
In vitro (glial culture)	3–10 replicates	Molecular specificity	Artificial conditions, no systemic interaction	Interpret as hypothesis-generating only

**Abbreviations:** PMS—progressive multiple sclerosis; CSF—cerebrospinal fluid; EAE—experimental autoimmune encephalomyelitis. **Notes:** Typical sample sizes represent approximate ranges from representative studies in the PMS literature. Bias modes indicate common methodological limitations inherent to each design.

**Table 3:** Summarized key glial markers associated with progressive MS.

Marker	Cell Type	Function	Relevance in PMS
Iba1	Microglia	Motility, phagocytosis	Marker of microglial activation; correlates with lesion expansion
CD68	Microglia	Lysosomal degradation	High expression in phagocytic microglia; associated with neurodegeneration
GFAP	Astrocytes	Cytoskeletal structure	Elevated in reactive astrocytes; contributes to glial scar formation
TSPO	Microglia/Astrocytes	Mitochondrial translocator	Imaging biomarker for glial activation (e.g., via PET radioligands)

**Table 4:** Major cytokines and molecular mediators in PMS.

Molecule/ Marker	What It Is	Primary Producing/ Expressing Cells in CNS	Role in Progressive MS (PMS)	Biomarker/ Assay Context	Notes	Study Design	Human/ Animal
TNF-α (TNFSF2)	Pro-inflammatory cytokine that signals via TNFR1 (death/inflammatory) and TNFR2 (repair/regulatory) receptors.	Microglia, astrocytes; infiltrating myeloid cells and T cells.	Chronically elevated in lesions/CSF; promotes glial activation, BBB permeability, and oligodendrocyte injury; TNFR1-associated neurotoxicity vs. TNFR2-associated repair.	Measured in CSF/serum (ELISA); lesion immunohistochemistry; single-cell transcriptomics in active rims.	Non-selective anti-TNF can exacerbate MS; selective TNFR1 blockade and TNFR2 agonism are proposed strategies.	Both (human tissue/CSF + preclinical)	Both
IL-6 (IL6)	Pleiotropic cytokine signaling via IL-6R/gp130, activating JAK/STAT3.	Astrocytes, microglia; meningeal B/plasma cells.	Overproduced intrathecally; sustains astrogliosis via STAT3, supports B cell survival; associated with cortical demyelination.	CSF/serum ELISA; intrathecal synthesis indices; transcriptomic detection in meningeal aggregates.	IL-6 receptor blockade (e.g., tocilizumab) modulates pathway in related disorders; role in PMS under investigation.	Both (human cohorts + preclinical)	Both
IL-1β (IL1B)	Pro-inflammatory cytokine matured by NLRP3 inflammasome (caspase-1-dependent processing).	Microglia (inflammasome-activated), infiltrating macrophages; astrocytes under stress.	Amplifies glial feedback loops and cytokine crosstalk; contributes to demyelination and chronic lesion activity.	CSF levels (often low but detectable); lesion immunostaining; peripheral monocyte-priming assays.	IL-1 receptor antagonists (e.g., anakinra) and NLRP3 inhibitors are mechanistically relevant; CNS penetration and efficacy remain under study.	Both (human tissue/CSF + preclinical)	Both
COX-2 (PTGS2)	Inducible cyclooxygenase isoform, catalyzes prostaglandin synthesis.	Activated microglia/macrophages, astrocytes, neurons.	Amplifies glial inflammatory cascades via prostaglandin signaling, contributes to excitotoxicity.	IHC/ISH for PTGS2; PGE2 levels in CSF/serum.	Often upregulated at rims of chronic active lesions; target of selective COX-2 inhibitors.	Reviews + pathology context	Both
iNOS (NOS2)	Inducible nitric oxide synthase producing high-output NO.	Activated microglia and macrophages; reactive astrocytes.	Excess NO/peroxynitrite damages mitochondria and oligodendrocytes, sustaining chronic neuroinflammation.	IHC for NOS2; nitrotyrosine adducts; NO metabolites.	Upregulated by IL-1β, TNF-α, IFN-γ; limited CNS-penetrant inhibitors available.	Reviews + human lesion pathology	Both
ROS (Reactive oxygen species)	Oxidants (superoxide, H2O2, OH·) from NOX2/mitochondria.	Activated microglia/macrophages, mitochondria.	Drives oxidative injury to lipids, DNA, proteins; central to chronic lesion expansion.	Oxidative adducts (4-HNE, MDA), 8-OHdG, nitrotyrosine in CSF/tissue.	Oxidative stress is a hallmark of PMS pathology.	Human pathology + reviews	Both
Iron/Ferritin (FTH1/FTL)	Iron storage proteins; ferritin reflects cellular iron load.	Microglia (iron-laden in rim lesions), oligodendrocytes.	Iron catalyzes ROS formation (Fenton chemistry); contributes to microglial priming and chronic lesion activity.	QSM MRI for iron; ferritin in CSF/serum; histopathology.	Paramagnetic rim lesions correlate with iron accumulation.	Human pathology + MRI validation	Human
LC3 (MAP1LC3A/B)	Autophagosome membrane protein; LC3-II reflects autophagy activity.	Neurons, oligodendrocytes, astrocytes, microglia.	Altered autophagy affects oligodendrocyte/myelin integrity and glial stress responses.	IHC/Western blot for LC3-I/II; EM puncta counts.	Interpreted with p62 (accumulates when autophagy impaired).	Reviews + preclinical EAE	Both
p62 (SQSTM1)	Autophagy adaptor binding ubiquitinated proteins to LC3.	Neurons, oligodendrocytes, astrocytes, microglia.	Build-up indicates defective autophagic flux; links to inflammasome activation.	IHC/Western blot for p62 aggregates; co-stain with LC3.	Intersects with Nrf2 signaling; imbalance implicated in PMS.	Reviews + preclinical EAE	Both
Iba1 (AIF1)	Actin-binding protein upregulated in activated microglia.	Microglia; infiltrating macrophages.	Marks activated microglia forming paramagnetic rims; mediates cytoskeletal remodeling and phagocytosis.	IHC for Iba1 to quantify microglial activation.	Used as imaging and pathological biomarker of active lesion rims.	Microglia reviews + PET studies	Both
GFAP	Astrocyte intermediate filament (reactive astrogliosis).	Astrocytes.	Astrogliosis walls off inflammation but promotes scar formation and axonal repair inhibition.	IHC for gliosis; serum/CSF GFAP (Simoa).	Serum GFAP elevated in PMS; biomarker of disease progression.	Prospective cohort + review	Human
CD68	Lysosomal glycoprotein of phagocytes (macrophages/microglia).	Microglia and infiltrating macrophages.	Marks phagocytic, lipid-laden microglia at lesion rims; linked to chronic tissue breakdown.	IHC for CD68 in lesion rims; MRI-pathology correlations.	CD68+ rims co-localize with iron on QSM and predict chronic progression.	Human pathology + MRI-pathology validation	Human

This table summarizes key inflammatory- and glial-related molecules implicated in progressive multiple sclerosis (PMS). Functional descriptions are based on evidence from human pathology, CSF/serum biomarker studies, and preclinical models. “Study Design” indicates whether findings are derived from human tissue, clinical cohorts, animal models, or combined sources. “Evidence Tier” reflects the relative strength and consistency of available data (high = robust replication across multiple human studies; moderate = supportive but limited or primarily preclinical evidence). Abbreviations: TNF-α, tumor necrosis factor-alpha; IL, interleukin; iNOS, inducible nitric oxide synthase; ROS, reactive oxygen species; COX-2, cyclooxygenase-2; LC3, microtubule-associated protein 1 light chain 3; p62, sequestosome-1; Iba1, ionized calcium-binding adapter molecule 1; GFAP, glial fibrillary acidic protein; CD68, cluster of differentiation 68.

**Table 5:** Evidence-tier map for central cytokine claims in PMS.

Cytokine/Mediator	Central Claim	Evidence Tier	Sample Sizes (Approx.)	Anchor Citations
TNF-α	Chronically elevated in PMS lesions; drives glial activation and oligodendrocyte injury	Strong human (post-mortem + CSF studies)	*n* ≈ 120 (lesion cohorts); *n* >200 (CSF biomarker)	Brambilla 2011 [38]; Olmos 2014 [37]; Lassmann 2012 [6]
IL-6	Intrathecal elevation linked to cortical atrophy and meningeal inflammation	Supportive human (CSF + autopsy); reinforced by preclinical	*n* ≈ 150	Navikas 1996 [44]; Howell 2011 [43]; Reali 2020 [41]
IL-1β/NLRP3	Upstream driver of TNF-α/IL-6; persistent inflammasome expression in PMS	Preclinical + post-mortem support only	*n* < 40 human autopsy; EAE models	Barclay 2020 [45]; Brambilla 2011 [38]
GFAP	Serum/CSF levels predict disability progression	Strong human evidence	*n* > 1000 pooled across cohorts	Freedman 2024 [13]
NfL	Non-specific neuroaxonal injury marker	Strong but non-specific human evidence	*n* > 3000 (pooled)	eBioMedicine 2024 guidance [13]
TSPO-PET	Detects microglial activation; predictive of later atrophy	Supportive human longitudinal	*n* ≈ 120	Marjo 2025 [14]; Guido 2025 [21]

Abbreviations: PMS—progressive multiple sclerosis; CSF—cerebrospinal fluid; TNF-α—tumor necrosis factor-alpha; IL—interleukin; GFAP—glial fibrillary acidic protein; NfL—neurofilament light chain; TSPO-PET—translocator protein positron emission tomography; QSM—quantitative susceptibility mapping; PRL—paramagnetic rim lesion. Evidence tiers were assigned pragmatically based on consistency and replication across study types: strong human evidence (≥2 independent in vivo cohorts or meta-analyses), supportive human evidence (single cohort or combined with autopsy findings), preclinical support only (animal or ex vivo models without consistent human replication), in vitro only (cell culture studies). Sample sizes are indicative ranges from representative studies and not exhaustive.

**Table 6:** Fluid and imaging biomarkers of progressive multiple sclerosis.

Biomarker	Modality	Cellular Source	Clinical Relevance
GFAP	Fluid (CSF/serum)	Astrocytes	Marker of astrogliosis; correlates with spinal cord atrophy and disease progression
Neurofilament light chain (NfL)	Fluid (CSF/serum)	Neurons/axons	General marker of neuroaxonal injury; widely used in clinical trials
sTREM2	Fluid (CSF)	Microglia	Reflects microglial activation and phagocytic function
CD14	Fluid (CSF)	Monocytes/microglia	Marker of innate immune activation; associated with inflammatory disease progression
IL-6, IL-8, CXCL13	Fluid (CSF)	Activated glia, lymphocytes	Elevated in PMS; correlate with cortical atrophy and disease activity
TSPO-PET	Imaging	Microglia, astrocytes	Visualizes glial activation; increased uptake in PMS lesions and deep gray matter
QSM/SWI MRI	Imaging	Iron-retaining microglia	Detects paramagnetic rim lesions; identifies chronic active plaques

GFAP—glial fibrillary acidic protein; NfL—neurofilament light chain; CSF—cerebrospinal fluid; sTREM2—soluble triggering receptor expressed on myeloid cells 2; TSPO—translocator protein; PET—positron emission tomography; QSM—quantitative susceptibility mapping; SWI—susceptibility-weighted imaging.

**Table 7:** Summary of emerging therapies for progressive multiple sclerosis.

Therapeutic Agent	Primary Target	Mechanism of Action	Clinical Development Stage	CNS Penetration
Metformin	AMPK pathway, autophagy	Enhances mitochondrial function, restores autophagic flux, suppresses IL-6/STAT3 signaling in glial cells	Repurposed; Phase II trials in PMS	Yes (confirmed CNS activity)
Tolebrutinib	Bruton’s tyrosine kinase (BTK)	Modulates CNS-resident microglia and peripheral B cells; reduces glial inflammation	Phase III clinical trials	Yes (designed for CNS penetration)
Deferiprone	Microglial iron overload	Chelates excess iron, limits ROS production via inhibition of Fenton chemistry	Early-phase; off-label in neurodegeneration	Partial (limited BBB permeability)
Rapamycin	mTOR pathway	Enhances autophagic clearance, reduces astrocyte reactivity, supports proteostasis	Preclinical; limited human trials	Variable (CNS effects demonstrated in models)
Dimethyl Fumarate	Nrf2 activation	Activates antioxidant defenses, reduces ROS-mediated injury and glial priming	Approved for RRMS; under PMS evaluation	Yes (clinically proven CNS penetration)
Anakinra	IL-1 receptor antagonist	Inhibits IL-1β signaling and inflammasome activity	Approved for systemic diseases; early CNS studies	Low (limited BBB crossing)
Tocilizumab	IL-6 receptor blockade	Suppresses IL-6/STAT3 pathway, reduces reactive astrogliosis	Phase I-II in neuroinflammation	Low–Moderate (limited CNS access; variable results)

Abbreviations: AMPK—AMP-activated protein kinase; BTK—Bruton’s tyrosine kinase; mTOR—mechanistic target of rapamycin; ROS—reactive oxygen species; Nrf2—nuclear factor erythroid 2-related factor 2; RRMS—relapsing–remitting multiple sclerosis; STAT3—signal transducer and activator of transcription 3. Clinical development stages are current as of mid-2025 and based on available data from registered clinical trials and translational literature.

**Table 8:** Comparative analysis of key cytokines and molecular pathways shared between PMS and other neurodegenerative diseases.

Molecule/Pathway	Other Diseases Involved	Mechanistic Similarity	Key Distinction in PMS	Therapeutic Implication
IL-6	AD	STAT3 activation, astrocyte-driven inflammation	CNS-compartmentalized IL-6 from astrocytes	IL-6 receptor inhibitors may be applicable
COX-2	AD, PD	Glial inflammation, synaptic dysfunction	Requires CNS-penetrant inhibitors due to chronic activation	COX-2 inhibitors may need reformulation for CNS access
TNF-α	ALS	Cytokine-mediated inflammation, peripheral in ALS	Produced mainly by CNS-resident microglia in PMS	Anti-TNF therapy limited; TNFR2 agonism is an alternative
Iron/ROS	PD, ALS	Oxidative stress via Fenton chemistry	Chronic iron retention and efflux dysfunction in microglia	Targeted CNS iron chelation strategies required
Autophagy (LC3, p62)	PD, AD	Impaired clearance of damaged proteins	Glial priming and mitochondrial dysfunction tightly linked	Metformin and rapamycin show promise across diseases

Abbreviations: PMS—progressive multiple sclerosis; AD—Alzheimer’s disease; PD—Parkinson’s disease; ALS—amyotrophic lateral sclerosis; ROS—reactive oxygen species; CNS—central nervous system; STAT3—signal transducer and activator of transcription 3; TNFR2—tumor necrosis factor receptor 2; LC3—microtubule-associated protein 1 light chain 3; p62—sequestosome 1.

## Abbreviation

PMS	Progressive Multiple Sclerosis
RRMS	Relapsing–Remitting Multiple Sclerosis
MS	Multiple Sclerosis
CNS	Central Nervous System
TNF-α	Tumor Necrosis Factor-alpha
IL-6	Interleukin-6
IL-1β	Interleukin-1 beta
IFN-γ	Interferon gamma
iNOS	Inducible Nitric Oxide Synthase
COX-2	Cyclooxygenase-2
ROS	Reactive Oxygen Species
TSPO	Translocator Protein
PET	Positron Emission Tomography
MRI	Magnetic Resonance Imaging
NLRP3	NOD-, LRR- and pyrin domain-containing protein 3
GFAP	Glial Fibrillary Acidic Protein
NfL	Neurofilament Light Chain
BBB	Blood–Brain Barrier
DMT	Disease-Modifying Therapy
TLR	Toll-like Receptor
MAPK	Mitogen-Activated Protein Kinase
NF-κB	Nuclear Factor kappa-light-chain-enhancer of activated B cells
JAK/STAT	Janus Kinase/Signal Transducer and Activator of Transcription
mTOR	Mechanistic Target of Rapamycin
GSH	Glutathione
ATP	Adenosine Triphosphate
ISI	Institute for Scientific Information
SANRA	Scale for the Assessment of Narrative Review Articles
